# Principles of the Treatment of Gout

**Published:** 1835-07-01

**Authors:** 


*Principles of the Treatment of Gout; with a further Examination of the Effects of Colchicum as a Remedy: and some Observations on the Use of Veratria in that Disease.* By Sir Charles Scudamore, m.d., f.r.s. London 1835. 8vo. pp. 54.

Sir C. Scudamore hopes that he "may, without incurring the charge of vanity and presumption, confidently present to the reader a code of principia, or principles, which may serve as a safe guidance to the knowledge and treatment of the disease." (p. 2.) Now, great as is the good humour of readers and reviewers in the present day, we are afraid that so tiny a code will not be deemed sufficient, in so wide and perplexed a subject as the treatment of gout. Were physicians absolute monarchs indeed, a few laws might suffice for the extirpation of gout. The spinage diet, with which Rust cured his obstinate case of chorea, or the apple fare with which Hufeland successfully combated an impetigo, might dispense with the necessity of colchicum or veratria: but, as Earls and Aldermen still refuse to live on a shilling a day, and work for it, physicians must still weave a rope of sand, while they endeavour to make health, and turtle, and a gouty habit, and colchicum, and champagne, cohere.

In the actual dearth, however, of fresh information on the subject, we will give an abstract of Sir C. Scudamore's opinions.

*Bleeding* is to be practised as a remedy for gout, only when general plethora is very manifest, and the pulse is strong; though, of course, a gouty patient may require bleeding for some other disease. Leeches are rarely useful in acute gout.

*Emetics* are generally beneficial at the commencement of a severe attack, especially if well-marked dyspepsia be present.

*Cathartics* are indicated by theory, and recommended by practice. A combination of calomel and cathartic extract agrees with most persons, and a small quantity of James's powder may often be added with advantage: or the mercurial dose may be given at night, with opium or extract of poppies, followed by an aperient in the morning, which may be strong or weak, cordial or saline, according to the case. Sir C. Scudamore falls into the error of saying, "It is incumbent on us to persist in acting with freedom on the bowels from day to day, so long as the alvine discharges exhibit an unnatural character, more particularly as to darkness of colour, which marks a vitiated state of the biliary secretion;" &c. (P. 13.) Surely patients who are taking mercury or colchicum, or both, cannot be expected to have natural stools: if purgatives are to be given to cure an appearance caused by purgatives, where is the medicinal cycle to end? This error has often been committed with regard to mercury, and is ably exposed in a passage of Dr. Armstrong's Lectures, which we quoted in our third number.

*Mercurials* are not to be given so as to produce salivation.

*Diuretics* are indicated by the deep colour of the urine; and the lateritious sediment which it deposits. Our author recommends a draught containing the carbonate of potash with the Sp. Æth. Nitr. and Sp. Junip. C. He also advises "the draught with magnesia, sulphate of magnesia, acetum colchici, &c., the formula of which will be found in my treatise." (P. 15.) This is not so well, Sir C. Scudamore, to send us to your treatise for the explication of the "&c.;" for it was an understood thing that the present "code of principia, or principles," was to supersede the treatise for practical purposes, and be the gouty man's Iliad in a nutshell.

*Sudorifics* are very useful. Tartar emetic may be combined with the aperients, or with saline draughts, or small and repeated doses of Dover's powder may be given. If it is difficult to cause diaphoresis, the body may be fomented with flannel wrung out of hot water, or with a large sponge. Much benefit is sometimes obtained from a vapour-bath, administered in the patient's chamber.

*Narcotics* may be given at night, combined with sudorifics, as Battley's sedative liquor, with tartarized antimony; opium, with James's powder; or Dover's powder. The acetate and muriate of morphia are excellent remedies. Sometimes, if the inflammation runs high, opiates will not calm the pain, until one or more bleedings have been premised.

*Colchicum.* Sir C. Scudamore prefers the acetum colchici to any other preparation; "joining with it magnesia, or the carbonate, and usually, sulphate of magnesia, as I fully set forth in my Treatise," (again referring his half-crown readers to his twenty shilling book.) The dose is from twenty to ninety minims, equal respectively to about one and five minims of the wine; the colchicum wine being seventeen times as strong as the vinegar.

Our author thinks that the preparations of colchicum have been used far too freely; one or two fits of the gout may be safely cured by it, but not so a dozen; and, when the intervals between the attacks become shorter and shorter under the use of the remedy, its continual administration will ruin the finest constitution. The following instances will show the injurious effects of colchicum; but our readers will observe, that, in all of them, the patients undertook the management of their own cases.

"One gentleman, of strong frame, and of great original powers of constitution, constantly suppressed his gout with one or other of the strong preparations of colchicum; latterly attaching himself to the use of the wine. This practice at length brought on chronic inflammation of the mucous membrane of the stomach and bowels; and, under such disease, he pined and wasted, and gradually lost strength, and died.

"Another, also of excellent constitution originally, not yet fifty years of age, treated himself in the same manner, but with greater boldness, and gave occasion to a more acute attack upon the mucous membrane. The highest symptoms of irritation, affecting the stomach and intestines, arose: and, notwithstanding every exertion to arrest the disease, the fever and irritation wore out the injured powers of the constitution, and death took place in the course of a fortnight. This gentleman had been falling off in appearance, and flesh, and strength, for some months previously, under the influence of almost constant doses of the wine of colchicum. He was warned of his danger, but to no purpose.

"A third, approaching to his grand climacteric, a man endowed with a fine constitution, was so anxiously engaged in business, that he would not allow himself to be deprived of the means of procuring that quick respite from suffering and confinement, which the wine of colchicum afforded him. He had recourse to this medicine on every occasion, beginning with a dose of thirty drops; but by degrees so increasing it, that at length he found he could not relieve the symptoms with a smaller dose than 120 drops, which he commonly repeated on the following night. Even by means of these larger doses, he could not latterly postpone the return of gout for more than forty-eight hours! His altered appearance soon gave alarm to his friends, and had he not been restrained from this course of proceeding, he would doubtless have fallen a victim. His health has been with difficulty brought to a state of amendment; but I hope that, by strict perseverance in proper measures, he will finally be restored.

"One gentleman took Reynolds' specific, in alterative doses, almost every night for a whole year. He became thin, sallow, weak, and nervous, under its influence, on such terms only opposing the return of gout. But see the sad sequel. At the end of the fourteenth month, he died from hydrothorax!

"In another case, the patient, aged fifty-four, for many years subject to severe attacks of regular gout, had for the last two years treated himself with full doses of the wine of colchicum, always with the effect of immediately relieving the symptoms, but with the effect also of causing much shorter intervals between the fits. At length his general health declined; his appearance became greatly changed; he looked sallow, was wasted, and reduced in strength. His pulse was weak and irregular; his breathing short, and he was awoke in the night with starting up in bed under the immediate dread of suffocation; and had a troublesome cough. Upon examination of the chest, it was evident that water was collected in one cavity of the pleura. Other frightful symptoms came on, affecting the heart, and the signs of enlargement of that vital organ became very conspicuous. He had urgent palpitations; could not lie down in bed; was now and then delirious while sitting in his easy chair, and it was continually expected that he would die from suffocation, so difficult was the breathing on the least exertion. The pulse ranged from 120 to 150. General anasarca of the lower part of the body took place in an extreme degree, and, lastly, ascites. By auscultation, the action of the heart was heard over the upper part of the right side, and below the seventh rib on the left, and the bruit du sufflet (bellows' sound) and the rasp-file sounds, were strongly marked. From this deplorable condition he was surprisingly restored by an active plan of treatment, which my present limits will only allow me to describe in the most concise manner. It consisted of two or three small bleedings from the arm; a combination of elaterium, calomel, digitalis, colocynth, and opium; blisters over the heart, followed by the use of an ointment composed of iodine, hydriodate of potash, and mercury. This was afterwards used by diligent friction. The anasarca was relieved by acupuncturation. He gradually recovered from what appeared to be a hopeless state. On the discontinuance of the active measures, a caustic issue was made over the heart, and which is still kept up. Ten months have now elapsed since the patient has resumed an active attention to his affairs, as a nobleman's steward. Now the action of the heart is almost natural. The recovery is the most surprising of the kind which I have ever witnessed in the course of my professional life." (P. 28.

Our author observes, that the continued use of colchicum produces chronic irritation of the mucous membrane of the stomach and intestines, loss of appetite, and other bad symptoms; and asserts, that none of these consequences ever result from the moderate use of the acetum colchici, in combination with other appropriate remedies; but we should think that the mucous membrane would be perfectly safe if the vinum colchici were administered in doses of from one to five minims, being the equivalents to those of the acetum colchici recommended by our author. It often happens, however, that the different preparations of the same remedy are not administered in equivalent doses. Thus one eighth of a grain of corrosive sublimate is a common dose; but two drachms of its solution are rarely given.

The following is a case of gout successfully treated without colchicum.

"A gentleman, about fifty years of age, had been a complete martyr to gout for twenty years; and, latterly, rarely escaped an attack for more than six weeks. He was so disturbed in his nervous system by the influence of colchicum, after a few doses, however carefully administered, that he acquired a great dread of the medicine, notwithstanding that it invariably relieved the symptoms in a short time. In the investigation of the case, I found the strongest evidence of unhealthy action of the liver. The excretions were exceedingly unnatural, varying for the most part from the colour of black to olive green. The urine usually deposited pink or lateritious sediment, and always very abundantly when gout was present.

"I subjected him to a moderate but long-continued alterative mercurial course, with an active fluid purgative each morning for the first week, each other morning for the second week, and every second or third morning for the third and fourth weeks. When a gouty attack occurred, I relieved the symptoms very satisfactorily by means of opiates, sudorifics, and diuretics, in addition to the plan just mentioned. At a later period, I prescribed sarsaparilla with alkali; still directing a close attention to the state of the alimentary canal.

"The whole course of treatment being brought to a conclusion, the patient left home for change of air, first going to the sea-side, and afterwards to an inland spot. He virtuously adhered to the strict plan of diet which was laid down for his observance; and such has been his reward, that, during the last six months, he has only been affected with gout twice in a slight degree, and the symptoms yielded favourably without any aid from colchicum. It is delightful to witness the happy change in this gentleman's appearance and constitution. He assures me that he has not enjoyed such feelings of health before within the last six years." (P. 38.)

In another case, "I was called upon to prescribe any means of treatment I thought proper, with the exception of colchicum;" the patient's constitution having been broken down by the remedy. Alteratives, aperients, tonics, and change of air, were successful here likewise.

Sir C. Scudamore remarks that

"One of the most inconvenient consequences arising to the physician from the colchicum practice, as it may be termed, is the extreme reluctance of the patient to submit himself to the slower but more sure effects of regular treatment. Accustomed, as he has been, to liberate himself from pain and confinement in the short space of not many hours, he refuses his consent to measures which are attended with a continued confinement; but I hope that this mistaken calculation of advantages will cease to be made, and the truth come home to his mind, that there is no such short road to health, as he has been falsely led to imagine." (P. 42.)

Our author has used the veratria ointment with some success: it is not, however, to be employed at the beginning of an attack, but when the force of the disease has been reduced by general treatment. In one case where the strength of the remedy was gradually increased till the proportion of the veratria was twenty grains to the ounce of lard, "the patient finally declared that he derived very great relief and benefit from the remedy; but that it did not remove the tenderness, and what he called weakness, of the parts. The bursal enlargements, the distension of the tendonous sheaths, and the ligamentous thickenings, were evidently diminished by the application; but it appeared ineffectual towards restoring the use of the joints. In these circumstances, abundant friction with an embrocation, composed of tinct. opii, linim. sapon. compos. et liquor. ammon. acet. used tepid, had a superior and a very useful effect." (P. 49.)

This little book is rather carelessly written. At p. 6, for instance, we are told that certain unnamed ancients of a later date than Hippocrates and Aretæus speak of the use of *calomel* in gout; and, at p. 21, the author says, that "a few, but, indeed they constitute the large minority, are attacked with so slight and manageable a degree of gout," &c.

The essay, however, with all its faults, is the production of a practical man, and this must serve as an apology for the length of our analysis.

